# An Improved Method for Soil DNA Extraction to Study the Microbial Assortment within Rhizospheric Region

**DOI:** 10.1155/2014/518960

**Published:** 2014-09-15

**Authors:** Faria Fatima, Neelam Pathak, Smita Rastogi Verma

**Affiliations:** ^1^Department of Biosciences, Integral University, Lucknow 226026, India; ^2^Department of Biotechnology, Delhi Technological University, Delhi 110042, India

## Abstract

The need for identification of soil microbial community mainly depends on direct extraction of DNA from soil, a multifaceted environment that is a major pool for microbial genetic diversity. The soil DNA extraction procedures usually suffer from two major problems, namely, inappropriate rupturing of cells and contamination with humic substances. In the present study, five protocols for single type of rhizospheric soil were investigated and their comparison indicated that the inclusion of 120 mM phosphate buffered saline (PBS) for washing and mannitol in the lysis buffer allowed the processing of soil sample in minimal time with no specific equipment requirement. Furthermore, DNA purity and yield were also improved, which allowed the exploitation of genetic potential of soil microbes within soil sample thereby facilitating the amplification of metagenomic DNA. The effectiveness of methods was analyzed using random amplification of polymorphic DNA. The banding patterns revealed that both the abundance and the composition of indigenous microbial community depend on the DNA recovery method.

## 1. Introduction

The biodiversity of microbes within soil is significant for the maintenance of healthy soil because these microbes are involved in many vital functions like crucial cycles of C, N, P, formation of soil, toxin removal, and so on. Previously, studies on the development of microbial communities required the isolation of these microbes from soil sample by culture dependent techniques followed by a series test for phenotypic evaluation and their identification. However, the microbial diversity studies conducted in soil have been biased essentially due to the unculturability of many microbes. Specific media, which are used to culture microbes, are selective in nature and only subpopulations of microbes from environment sample that will grow mainly depend on the particular conditions. It is reported that only 1% of microbes can be cultured in the laboratory using traditional culture techniques [1].

To study the microbial community, microbiologists have adopted culture-independent techniques. These techniques employ molecular biology based methods, in which soil extracted nucleic acid is subjected to PCR amplification [2]. These methods provide a unique insight into richness, composition, and structure of microbial community, that is, species richness and species evenness. The results thus rely not only on DNA extraction procedures but also on the factors affecting PCR amplification. Moreover, these culture-independent methods should address the problems like incomplete rupturing of cells and presence of soil organic substances, namely, fulvic and humic acid, the presence of which inhibit the activity of DNA polymerase, and interfere with the hybridization protocols [3]. Fractions like humic acids are usually the complex mixtures of related compounds (DNA) demonstrating a broad range spectrum of solubility and charge characteristics. Various physical and chemical treatments have been evaluated for cell rupture, which include shaking the sample in lysis buffers containing high concentration of sand, detergents or glass beads, inclusion of lysozyme [4]. Furthermore, purification of silica and other biogel columns has been reported to minimize the humic acid contamination. These procedures, however, make DNA isolation process expensive involving a large number of steps, which makes these procedures lengthy, time-consuming, and tedious. Therefore, an improved method is required for soil DNA extraction that would allow efficient rupturing of microbial cells and simultaneously decrease the contamination of organic materials (humic acid) in an easy and cost-effective manner.

The analysis of microbial diversity in the soil DNA extracts is then based on ARDRA-amplified ribosomal DNA restriction analysis [5], DGGE-denaturing gradient gel electrophoresis, and “T”-RFLP-Transfer restriction fragment length polymorphism [6]. However, random amplification of polymorphic DNA (RAPD) technique is preferred, as the above described techniques may not amplify fragments from all community members with equal effectiveness. Such approach thus offers significant advantage over just 1% of the microbial community accessible with standard, culture-based techniques. An additional advantage is that only small amount of soil sample is required for analyzing microbial diversity in a short span of time.

In the present study, four DNA extraction methods and a commercial* Soil Master* DNA extraction kit were used to extract DNA directly from soil and the effectiveness of these methods was estimated by RAPD analysis.

## 2. Material and Methods

### 2.1. DNA Extraction Methods

Five DNA extraction methods were evaluated in this study with respect to the quality and purity of extracted DNA using single type of rhizospheric soil. Three modified mannitol-based methods [7], polyethylene glycol (PEG/NaCl) method [8], and a soil DNA extraction kit were compared for obtaining a high recovery and DNA with good yield and purity. Isolated soil samples were isolated and immediately placed on dry ice, mixed, and then stored at −20°C prior to DNA extraction.

#### 2.1.1. DNA Extraction Using Polyethylene Glycol (PEG)/NaCl Method

One gram of soil sample was mixed with 10 mL of DNA extraction buffer (120 mM Na_2_HPO_4_ (pH 7.4), 5% SDS (w/v) and 0.02 g PVPP) in centrifuge tubes and incubated for 1 h at 65°C with occasional stirring. The supernatant was collected after centrifugation at 8,000 rpm for 10 min at 4°C and mixed with half volume of PEG and 1 volume of NaCl and incubated at 4°C for overnight. Further, 1 volume of chloroform : isoamyl alcohol (24 : 1) was added and centrifuged at 12,000 rpm for 10 min at 4°C. The supernatant obtained was precipitated by addition of 1/10th volume of 3 M sodium acetate (pH 5.2) and 2 volumes of ethanol. Finally, the pellet was recovered by centrifugation at 12,000 rpm at 4°C and dissolved in 25 *μ*L TE buffer (Tris-HCl 10 mM (pH 7.8); EDTA 1 mM (pH 8)).

#### 2.1.2. DNA Extraction Using Soil DNA Extraction Kit (*Soil Master* DNA Extraction Kit)

DNA was extracted from soil sample (1 gm) according to the specifications of the supplier (EPICENTRE, Madison, WI, USA). The method involved direct cell lysis with prewarmed 5 mL solution A at 65°C and vortexing for 10 min. This mixture was incubated for 15 min at 65°C. The supernatants were collected after centrifugation at 8,000 rpm at 4°C for 10 min and mixed with equal volume of solution B that led to precipitation. The steps were repeated two times when the color of supernatant changed to yellow. Finally the pellet was recovered by centrifugation at 12,000 rpm for 10 min at 4°C and dissolved in 25 *μ*L TE buffer.

#### 2.1.3. Modified Mannitol-Based Methods

One gram of soil sample was ground using liquid nitrogen. This was followed by addition of 5 mL of 120 mM phosphate buffer saline (pH 7.4) and shaking at 150 rpm for 10 min at 4°C. The soil suspension was centrifuged at 7,000 rpm for 10 min. The pellet was rewashed with PBS buffer and suspended in 10 mL of DNA extraction buffer containing 1 M Tris-HCl (pH 8.0), 5 M NaCl, 0.5 M EDTA (pH 8.0), 10% CTAB, 10% SDS, and 0.2 M mannitol. The suspension was incubated for 1 h at 65°C with occasional stirring of 150 rpm and subjected to three different treatments as indicated.

*(i) DNA Extraction by Mannitol-Phosphate Buffer Saline-Polyethylene Glycol/Sodium Chloride (Mannitol-PBS-PEG/NaCl) Method*. The soil suspension described above was centrifuged at 8,000 rpm for 10 min at 4°C and the supernatant obtained was mixed with half volume of polyethylene glycol-8000 (50%) (PEG) and 1 volume of NaCl and allowed to incubate at 4°C for overnight. The pellet was recovered by centrifugation at 12,000 rpm at 4°C for 10 min and dissolved in 3 mL of TE buffer. The DNA sample was then purified by phenol : chloroform extraction. Finally DNA was precipitated by addition of 1/10th volume of 3 M sodium acetate (pH 5.2) and 2 volumes of ethanol. The pellet was recovered by centrifugation at 12,000 rpm for 10 min at 4°C and dissolved in 25 *μ*L TE buffer (Tris-HCl 10 mM (pH 7.8); EDTA 1 mM (pH 8)).

*(ii) DNA Extraction by Mannitol-Phosphate Buffer Saline-Phenol/Chloroform/Isoamyl Alcohol (Mannitol-PBS-PCI) Method*. After centrifuging the soil suspension as previously described, the supernatant thus obtained was extracted with an equal volume of PCI by centrifugation at 12,000 rpm for 10 min at at 4°C. Aqueous fraction was mixed with 1/10th volume of 3 M sodium acetate (pH 5.2) and 2 volumes of 70% chilled ethanol at 4°C. Finally the pellet was recovered by centrifugation at 12,000 rpm for 10 min at 65°C and dissolved in 25 *μ*L TE buffer (Tris-HCl 10 mM (pH 7.8); EDTA 1 mM (pH 8)).

*(iii) DNA Extraction by Mannitol-Phosphate Buffer Saline-Cetrimide (Mannitol-PBS-CTAB) Method*. After centrifugation of soil suspension, 50 *μ*L of 5 M NaCl and 50 *μ*L of 10% CTAB (cetrimide prepared in 0.7 M NaCl) were added to the supernatant and incubated at 4°C for 15 min. This was followed by addition of equal volume of PCI and centrifugation at 12,000 rpm at 4°C overnight. Aqueous layer was allowed to precipitate overnight at 4°C with 1/10th volume of 3 M sodium acetate (pH 5.2) and 2 volumes of ethanol. Finally the pellet was recovered by centrifugation at 12,000 rpm for 10 min at 65°C and dissolved in 25 *μ*L TE buffer (Tris-HCl 10 mM (pH 7.8); EDTA 1 mM (pH 8)).

### 2.2. DNA Yield and Purity

The DNA concentration of the soil sample was measured by examining the absorbance of the sample at 260 nm and the amount of DNA was calculated (1.0 A_260_ unit = 50 *μ*g/mL of DNA) [8]. The purity of the extracted DNA was determined by taking absorbance at 230, 260, and 280 nm. A pure sample of DNA has the A_260_/A_280_ ratio as 1.8 and the A_260_/A_230_ ratio as 2.0, whereas DNA preparation that is contaminated with protein will have an A_260_/A_280_ ratio lower than 1.8 [9].

### 2.3. PCR Amplification of Isolated Soil DNA Using 16S rRNA Primers for Bacterial Identification

Soil DNA was amplified by PCR using a PCR BIORAD Thermal Cycler (United Kingdom). Each 25 *μ*L PCR mixture contained 1 *μ*L (1 : 10 dilution) community DNA (10 ng–20 ng), 2.5 *μ*L PCR buffer (1X), 1 *μ*L of each deoxyribonucleoside triphosphate (dNTP) (100 mM), 1 *μ*L of forward and reverse primers (0.5 *μ*M), and 0.5 *μ*L* Taq* polymerase (3 U) (Fermentas). The 16S rRNA regions were amplified by using 16S rRNA primers, namely, (FP1) 5′-TGGGGAGCAAACAGGATTAG-3′ and (RP1) 5′-TAAGGTTCTTCGCTTGCTT-3′. The amplification cycle consisted of an initial denaturation step of 30 min at 94°C, followed by 35 cycles of 1 min at 94°C (denaturation), 1 min at 55°C (annealing), and 2 min at 72°C (extension), with a final extension step for 5 min at 72°C. For visualizing PCR products, 5 *μ*L of the amplified product was electrophoresed on 1% agarose gel in 1X TAE buffer, stained with ethidium bromide (EtBr 0.5 *μ*g/mL) and analyzed by gel documentation system (BIORAD). Lambda DNA* Eco*RI/*Hin*d-III double digest was used as a molecular size marker.

### 2.4. PCR Amplification of Soil DNA Extract Using 18S rRNA Primers for Fungal Identification

Soil DNA was submitted for PCR amplification by using PCR BIORAD Thermal Cycler (United Kingdom). A region from 18S rRNA gene was amplified using internal transcribed spacer (ITS) primers, namely, ITS 5: (5′-GGAAGTAAAAGTCGTAACAAGG-3′) and ITS 4 (5′-TCCTCCGCTTATTGATATGC-3′). Each 25 *μ*L reaction mixture contained 2 *μ*L soil DNA (10 ng–20 ng), 2.5 *μ*L PCR buffer (1X), 0.5 *μ*L deoxyribonucleoside triphosphate (dNTP mix) (100 mM), 0.5 *μ*L of forward and reverse primers (0.2 *μ*M), 2 *μ*L of MgCl_2_ (25 mM), and 0.5 *μ*L* Taq* polymerase (3 U) (Fermentas). The amplification cycle consisted of an initial denaturation step of 5 min at 94°C, followed by 35 cycles of 1 min at 94°C (denaturation), 1 min at 59°C (annealing), and 2 min at 72°C (extension), with a final extension step for 10 min at 72°C.

### 2.5. Random Amplification of Polymorphic DNA (RAPD)

To test the efficiency of soil DNA extraction methods, RAPD was performed on community DNA. Four decameric RAPD primers, namely, OPA 3, OPA 13, OPA 15, and OPA 20 (Operon Technologies), were investigated (Table 1). Random primers are short DNA fragments of arbitrary nucleotide sequence that can differentiate between genetically distinct individuals. The RAPD analysis was carried out through PCR amplification of total DNA. Amplification reactions were performed in a total volume of 25 *μ*L containing 2.5 *μ*L PCR-buffer (10X), 2.5 *μ*L dNTP mix (2 mM), 1 *μ*L decameric primers (20 pmole), 2 *μ*L template (soil) DNA (100 ng), and 0.5 *μ*L* Taq* DNA polymerase (3 U). The final volume was made up to 25 *μ*L using sterile distilled water. The amplification reaction was performed for 45 cycles, and each cycle comprised of 3 min at 94°C (denaturation), 1 min at 32°C (annealing), and 2 min at 72°C (extension), with a final extension at 72°C for 10 min.

### 2.6. Analysis of PCR Products

For visualizing PCR products, 5 *μ*L of the amplified product was electrophoresed on 1% agarose gel in 1X TAE buffer, stained with ethidium bromide (0.5 *μ*g/mL), and analyzed by Gel Documentation system (BIORAD). Lambda DNA* Eco*RI/*Hin*d*-*III double digest was used as a molecular size marker.

## 3. Results and Discussion

For soil microbial analysis, it is essential to design protocols which yield high quality soil DNA of appropriate yield and purity for PCR amplifications. Besides, the selected methods for soil DNA extraction should be cost-effective and time-saving. Effectiveness of soil DNA extraction procedures may be influenced by various parameters such as incomplete cell lysis, DNA sorption to soil surfaces, extraction of humic contaminants, and DNA degradation. Thus, extraction of high molecular weight DNA, proper lysis of microbes, and inhibitor-free DNA are the major requirements for any protocol used for metagenomic study [10].

For cell lysis to be effective, mechanical treatment should be followed rather than chemical ones [11]. According to Frostegård et al. [4], proper grinding of sample ruptures the cell wall thereby releasing the cellular DNA from the inner compartment.

Soil DNA extraction procedures should therefore be free from PCR inhibitors or their concentration must be low enough so that they do not interfere with the enzymatic reactions. Usually organic matter is the major source of inhibitors that may be coextracted with the microbial DNA present with in the soil. Majorly, humic acids create considerable problem like interference in activity of DNA polymerase used for PCR reactions [12]. As humic acid contains the same charge and size characteristics like DNA, it exhibits absorbance at both 230 and at 260 nm and hence interferes in quantization of DNA. This characteristic can be used to find out the level of contamination of humic acid in an isolated DNA sample.

The present study involved comparison of five methods for isolation of soil DNA. Three methods having mannitol in their extraction buffer yielded an amount of DNA that was significantly higher than that obtained with the* Soil Master* DNA extraction kit and PEG/NaCl method (Table 2). Moreover, the purity of DNA isolated by modified mannitol-based methods was significantly higher as compared to other methods (Table 2). The addition of mannitol in the extraction buffer has already been reported to enhance the efficiency of soil DNA extraction [7]. Further modification of these methods by inclusion of 120 mM phosphate buffered saline in the initial steps led to reduction in the level of organic contaminants such as humic acid at initial stages (Table 2).* Soil Master* DNA extraction kit and PEG/NaCl method with liquid nitrogen method consistently extracted DNA with higher A_260/230_ and A_260/280_ ratios, thereby indicating that the DNA was contaminated with humic acid-like compounds and proteins, respectively. A_260/230_ ratio of more than 2.0 was obtained with all the three mannitol-based methods, which was indicative of the fact that humic acid material very effectively reduced by these methods as compared to the other two methods.

**Figure 1:**
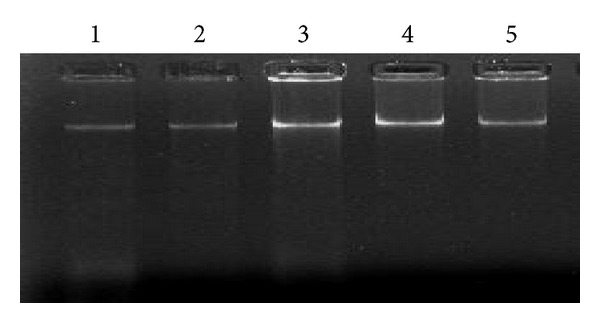
Visualization of soil DNA extracted by various methods. Lane 1: PEG/NaCl method without liquid nitrogen; lane 2:* Soil Master* DNA extraction kit; lane 3: mannitol-PBS-CTAB method; lane 4: mannitol-PBS-PCI method; lane 5: mannitol-PBS-PEG/NaCl method.

The three modified mannitol-based methods led to the recovery of high molecular weight soil DNA (Figure 1). The recovery of high molecular weight DNA fraction is desirable for PCR assays used for microbial diversity analysis because the degraded DNA upholds the formation of chimera products. Moreover, these three modified mannitol-based methods were found to be more suited for PCR amplification. Nuclear rRNA genes have been useful in the molecular study of bacterial and fungal diversity [13].

**Figure 2:**
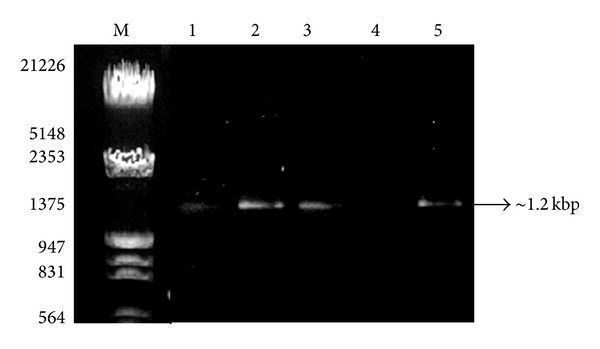
Visualization of PCR amplification products of soil DNA isolated by five different methods using 16S rRNA by different methods. Lane M: *λ* DNA* Eco*RI/*Hin*d III double digest marker; lane 1: mannitol-PBS-PCI method; lane 2:* Soil Master* DNA extraction kit; lane 3: mannitol-PBS-CTAB method; lane 4: PEG/NaCl method without liquid nitrogen; lane 5: mannitol-PBS-PEG/NaCl method.

**Figure 3:**
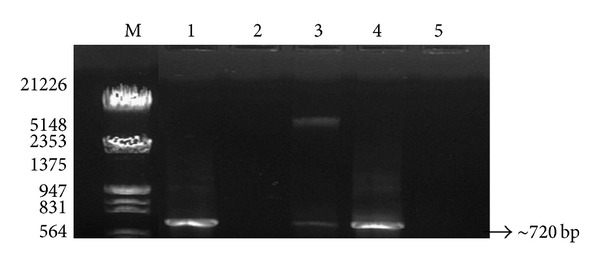
Visualization of PCR amplification products of soil DNA isolated by five different methods using 18S rRNA by different methods. Lane M: *λ* DNA* Eco*RI/*Hin*d III double digest marker; lane 1: mannitol-PBS-CTAB method; lane 2: PEG/NaCl method without liquid nitrogen; lane 3: mannitol-PBS-PEG/NaCl method; lane 4: mannitol-PBS-PCI method; lane 5:* Soil Master* DNA extraction kit.

High quality PCR amplicons with higher yields were observed in case of these three methods using 16S rRNA-specific and ITS-specific primers (ITS1/ITS4) for bacterial and fungal analysis, respectively. In each case, amplified products corresponded to expected sizes according to primers used. A single amplification product of ~1.2 Kbp for bacteria (Figure 2) and ~720 bp for fungi (Figure 3) was obtained. In case of the other two methods, namely, PEG/NaCl with liquid nitrogen method and* Soil Master* DNA extraction kit, an acceptable level of DNA was amplified for bacterial community (Figure 2; lanes 1 and 4) but these two methods were not suitable for PCR amplification for fungal study (Figure 3; lanes 2 and 5). Usually, an enzyme DNA polymerase used in amplification processes requires contamination-free sites for proper functioning. Furthermore, better PCR amplification of soil DNA isolated by the three modified mannitol-based methods demonstrated better DNA yield and quality as compared to the other two methods. The study suggested that all three mannitol-based methods (PCI, PEG/NaCl, and CTAB) gave very good yield of DNA which was suitable for the amplification study in comparison with the other two methods which might be due to DNA-adhering substances like humic/fulvic acid having the same charge characteristics as those of DNA that were coprecipitated in the mannitol-devoid methods. Humic acid impurities may affect DNA hybridization efficiency too. The method in which mannitol with liquid nitrogen was used, showed high-quality chemical lysis as compared to other methods. It also proves that an addition of PBS bufffer and mannitol may play an important role in proper chemical lysis of the cells as compared to other chemicals like CTAB, SDS, EDTA, and so forth.

Thus, these methods proved to be a low-cost and practical alternative to accessing metagenomic content by addition of phosphate buffer (PBS) and mannitol within the soil sample.

**Figure 4:**
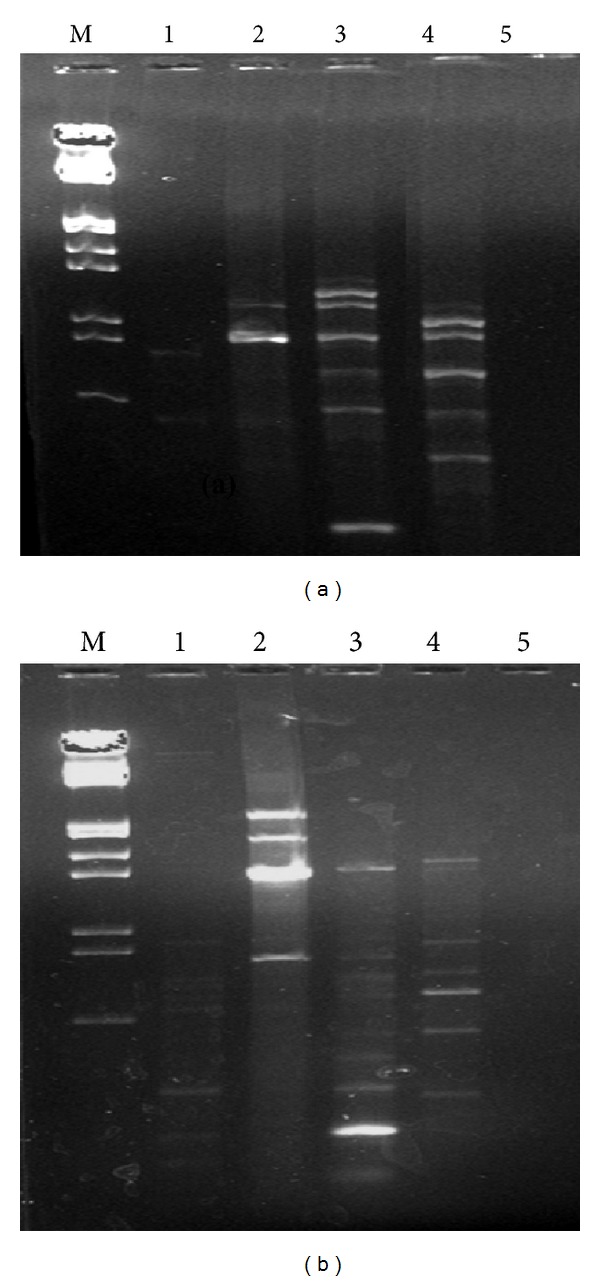
RAPD analysis of soil DNA samples isolated by five methods using random decameric primers. (a) OPA 3 and (b) OPA 13. Lane M: *λ* DNA* Eco*RI/*Hin*d III double digest marker; lane 1:* Soil Master* DNA extraction kit; lane 2: mannitol-PBS-CTAB method; lane 3: mannitol-PBS-PEG/NaCl method; lane 4: mannitol-PBS-PCI method; lane 5: PEG/NaCl method without liquid nitrogen.

**Figure 5:**
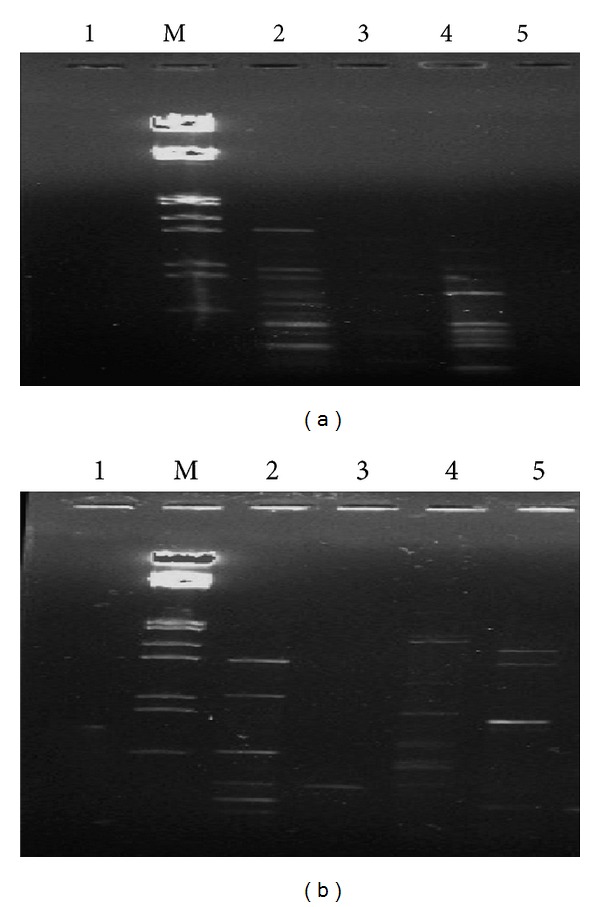
RAPD analysis of soil DNA samples isolated by five methods using random decameric primers. (a) OPA 15 and (b) OPA 20. Lane M: *λ* DNA* Eco*RI/*Hin*d III double digest marker; lane 1: PEG/NaCl method without liquid nitrogen; lane 2: mannitol-PBS-CTAB method; lane 3:* Soil Master* DNA extraction kit; lane 4: mannitol-PBS-PCI method; lane 5: mannitol-PBS-PEG/NaCl method.

Varying patterns of RAPD bands were found when soil community DNA samples were amplified using random primers. This indicated that the reported soil DNA extraction methods were quite feasible and reproducible for microbial diversity analysis (Figures 4 and 5). It was observed that the soil DNA isolated by protocols having mannitol was amplified easier than the methods using PEG/NaCl and soil DNA extraction kit.

Thus, the procedure presented here proves to be an inexpensive procedure, which not only prevents the loss of DNA but also reduces the risk of contamination by laboratory DNA source. The protocols involved the usage of mannitol within the lysis buffer to isolate DNA from bacterial and fungal mycelia. The methods used in the present study exhibited sufficient quality and integrity to amplify the genetic regions, which provided a complete information and understanding of microbial biota. An inclusion of mannitol and sodium chloride promoted cell disruption and extracted humic acid and other organic contaminants, the presence of which would have otherwise inhibited PCR reaction. Hereby, it is demonstrated that a molecular approach using culture independent study can be used to complement more traditional methods used for the survey of microbial communities and provides an expanding toolbox, which helps the soil ecologists and taxonomists to explore microbial communities, which are still unidentified. The modified protocols can also contribute to* in situ* study of bacterial and fungal ecological processes.

## 4. Conclusion

In the present study, efficient soil DNA extraction procedures have been reported, which are simple and efficient and do not require elaborate instrumentation and yield good quality DNA suitable for the study of bacterial and fungal genes. It has been demonstrated that an additional step of using phosphate buffer saline with inclusion of mannitol was useful to achieve these objectives. The PCR amplification procedures involve several enzymatic reactions where the enzyme DNA polymerase requires sites, which should be contamination-free. It is suggested that the initial washing with PBS buffer led to removal of unwanted impurities such as humic acid present in the soil. Mannitol, having high salt nature, led the recovery of high molecular weight DNA. It probably interacted with cell wall resulting in cell disruption and extraction of humic acid near the beginning of the isolation procedure. Thus, these modified mannitol-based protocols help not only in improving the yield and quality of extracted soil DNA but also in exploitation of large-scale preparations which provide greater possibility for detecting genes present in low abundance within the soil environment. When combined with the methods developed for normalization of total metagenomic DNA, these modified protocols may offer an easy method for monitoring the population dynamics of the total microbial population in soils over time.

## Figures and Tables

**Table 1:** Random primers used for RAPD analysis and their annealing temperature.

Random primer	Primer sequences	Annealing temperature
OPA 3	5′-AGTCAGCCAC	32°C
OPA 13	5′-CAGCACCCAC	32°C
OPA 15	5′-TTCCGAACCC	32°C
OPA 20	5′-GTTGCGATCC	32°C

**Table 2:** Comparison of amount, purity of DNA, and humic acid contamination extracted from various isolation protocols.

DNA extraction protocol	Amount of DNA (*µ*g/mL)	A_260/230_	A_260/280_
DNA extraction using PEG/NaCl method [8]	0.73	1.12	1.26
DNA extraction using commercial soil DNA extraction kit (*Soil Master* DNA extraction kit; EPICENTRE, Madison, WI, USA)	0.79	1.21	1.32
DNA extraction by mannitol-PBS-PEG/NaCl method	2.20	1.84	1.81
DNA extraction by mannitol-PBS-PCI method	2.36	1.93	1.84
DNA extraction by mannitol-PBS-CTAB method	2.67	2.07	1.85
